# 4124 An innovative Tool for Completing the Clinical and Translational Science Award (CTSA) Research Performance Progress Report (RPPR) using REDCap

**DOI:** 10.1017/cts.2020.229

**Published:** 2020-07-29

**Authors:** Maran Subramain, DeAnna O’Quinn, Heath Davis

**Affiliations:** 1 University Of Iowa Institute for Clinical and Translational Science; 2 University of Iowa

## Abstract

OBJECTIVES/GOALS: The RPPR Tool was created to accurately and systematically track our CTSA’s overall program goals and core’s progress in real time. It establishes and centralizes the continuous collection of key performance indicators and fosters accountability and transparency among cores and leadership. METHODS/STUDY POPULATION: Using the University of Chicago’s Annual Progress Report REDCap data dictionary, UI Institute for Clinical and Translational Science (ICTS) core managers convened to explore the adaptability of the reporting format for the CTSA. The team developed the more user friendly and easily accessible RPPR Reporting Tool using REDCap to better fit our CTSA. The RPPR in REDCap provides a central location to monitor the activities for each core, gather status updates, generate performance reports, and identify key performance indicators and challenges to prevent failures in the future. All data are transparent and accessible on-demand to individual core managers, evaluators, and ICTS leadership. RESULTS/ANTICIPATED RESULTS: UI’s RPPR Tool has improved the compliance with ongoing monitoring and reporting of CTSA program’s performance. Documenting all relevant information in a centralized space has eased the administrative and evaluation burden of preparing the RPPR. Furthermore, REDCap as a commonly used tool allows the core managers to complete this reporting with minimal guidance. This tool encourages each core to be accountable for documenting their respective progress. The transparency of the reporting allows the Co-PIs along with the leadership team to access the data at any given time to stay updated on the ICTS’ overall progress and to make the appropriate improvements as needed. DISCUSSION/SIGNIFICANCE OF IMPACT: The RPPR is a required component of all CTSA grants. UI’s RPPR Tool has been instrumental in comprehensively tracking progress of the ICTS and its contributions to translational research. UI is collaborating with CTSA peers to improve the RPPR Tool, so it can become an asset for any CTSA to adapt.

